# A Case of Liver Injury Immediately After Initiation of Triple Therapy in a Patient With BRAF V600E Mutation-Positive Colorectal Cancer

**DOI:** 10.7759/cureus.67424

**Published:** 2024-08-21

**Authors:** Yuka Aimono, Atsushi Ohkawara, Tatsunori Ogawa, Takahiro Yagisawa, Akihiro Tamura

**Affiliations:** 1 Pharmacy, Hitachi General Hospital, Hitachi, JPN; 2 Gastroenterology, Hitachi General Hospital, Hitachi, JPN

**Keywords:** pharmaceutical service, liver injury, cetuximab, encorafenib, binimetinib

## Abstract

The combination therapy of binimetinib, encorafenib, and cetuximab ("triplet regimen") was approved in Japan in November 2020 for the second-line treatment of BRAF V600E mutation-positive colorectal cancer. In this study, we encountered a patient who developed a liver injury requiring drug withdrawal on day eight of the triplet regimen administration. The protocol for the international phase III study did not specify blood and biochemical tests on day eight. Hepatic failure occurred in 45.9% (222/484) of the patients within three months, but no specific timing of onset was reported. Since it is important to disseminate novel adverse events to the public, we report the results of this study based on a literature review.

## Introduction

The combination of binimetinib (BINI), encorafenib (ENCO), and cetuximab (CET) (“triplet regimen”) was approved in November 2020 in Japan as a second-line treatment for BRAF V600E mutation-positive colorectal cancer. Although the onset of liver injury was mostly within three months in an international phase III trial, its exact timing remains unknown [1]. Among hepatic disorders, grade 3 or higher alanine aminotransferase (ALT) elevations were reported in 0.9% (1/222) and 0% (0/3) of Japanese patients [1]. However, there were no severe ALT elevations in post-marketing surveillance [2]. We herein report a case of an ALT elevation (grade 3) on the eighth day of drug administration that necessitated withdrawal.

## Case presentation

We present a male in his 40s with T4aN3M1b stage IVb sigmoid colon cancer (KRAS mutation-negative, BRAF mutation-positive, and MSI negative). He had a history of lumbar spondylolisthesis and his current medical history included a colostomy in March 2019 and a CV port one month later. Primary treatment with fluorouracil, leucovorin, oxaliplatin, and irinotecan (FOLFOXIRI) plus bevacizumab was initiated; however, due to worsening neurological symptoms, the patient was treated without oxaliplatin from the ninth course. In January 2022, the patient was judged to have progressive disease after 51 courses and, thus, was admitted in February for a triplet induction regimen. The main results of blood biochemical tests at admission are shown in Table 1.

**Table 1:** Laboratory findings of the patient. The main results of blood biochemical tests at admission. PLT: platelet; T-bil: total bilirubin; AST: aspartate aminotransferase; ALT: alanine aminotransferase; GGT: γ-glutamyltransferase; BUN: blood urea nitrogen; Cre: creatinine; CEA: carcinoembryonic antigen; CA19-9: carbohydrate antigen 19-9

Investigation	Reference values	Units	On admission	Day 8	Day 15	Day 22
WBC	3500~9000	Per μL	4,800	7,400	6,300	9,200
Hb	13.5~18.0	g/dL	15.6	15.8	15.7	15.3
PLT	12.5~37.0×10^3^	Per μL	21.5×10^3^	24.6×10^3^	28.7×10^3^	26.7×10^3^
T-bil	0.2~1.0	mg/dL	0.8	0.5	0.9	0.5
AST	7~38	U/L	23	102	28	36
ALT	8~43	U/L	18	223	68	44
GGT	0~50	U/L	44	287	208	122
BUN	6.0~20.0	mg/dL	16.8	22	12.2	16.8
Cre	0.6~1.2	mg/dL	0.86	0.91	0.8	0.96
CEA	0.0~3.4	ng/mL	2	-	-	-
CA19-9	0~37	U/mL	2 >	-	-	-

**Figure 1:**
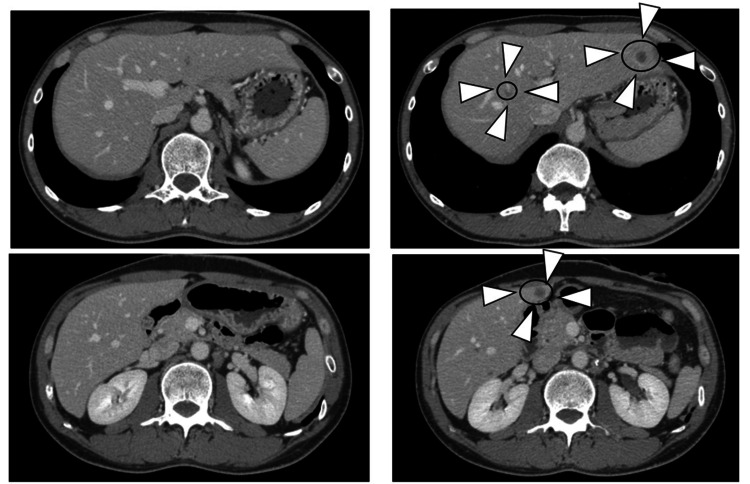
Abdominal CT findings of the patient. Comparison between four months before (left) and at the starting of the triplet regimen (right). Multiple liver metastases appeared and some lymph node metastases increased in size.

Abdominal CT findings, as shown in Figure 1, revealed numerous enlarged lymph nodes with calcification in the sigmoid mesentery, para-aortic region, and left supraclavicular fossa, which were considered to be lymph node metastases. The sizes of the para-aortic area and other areas had increased from four months earlier, and multiple liver metastases had also appeared.

**Figure 2:**
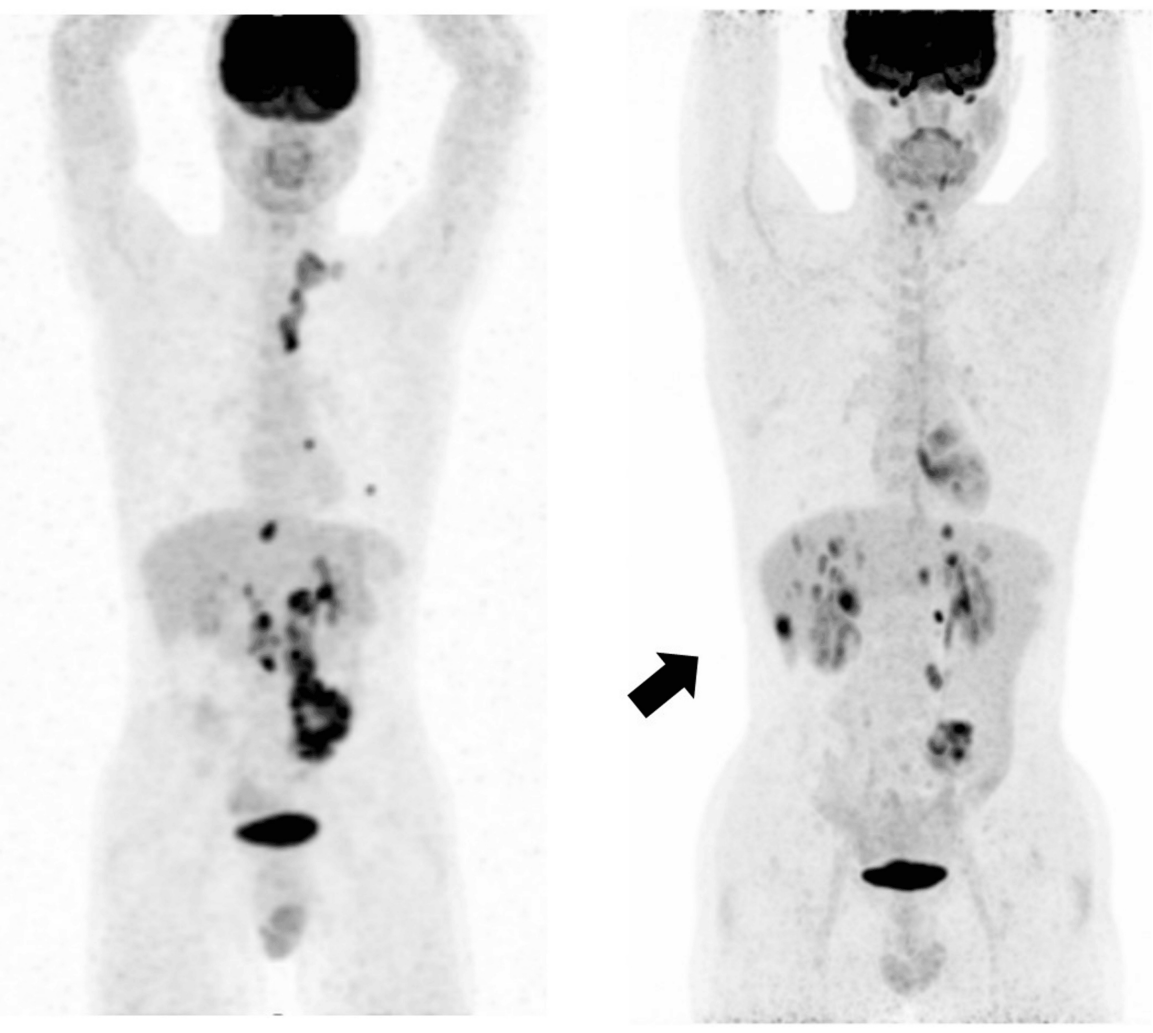
PET/CT findings of the patient. Residual abnormal accumulation was observed in the SD junction with SUV max 13.08, para-aortic lymph node 11.53, and mesenteric lymph node 7.54. Multiple liver metastases were observed, including SUV max 10.87 in S4, 9.39 in S2, and 7.67 in S8. SUV: standardized uptake value

PET/CT findings showed residual abnormal accumulation in the SD junction with standardized uptake value (SUV) max 13.08, para-aortic lymph node 11.53, and mesenteric lymph node 7.54. Multiple liver metastases were observed, including SUV max 10.87 in S4, 9.39 in S2, and 7.67 in S8 (Figure 2). Pathological findings revealed that the entire lesion comprised mucus, necrotic tissue, and fibrous tissue. Mucus-producing adenocarcinoma, growing in a cribriform or agglomerated manner, was suspended within the mucus.

Clinical course

Visual field disturbance appeared at noon on the triplet regimen day one. An examination by an ophthalmologist revealed no abnormalities of the eyelids or conjunctiva. On the other hand, serous pigment epithelial detachment and edema were detected in both macular areas, and kallidinogenase (kall) was prescribed. The patient was discharged on day four with no other adverse events. The results of blood biochemical tests on day eight showed liver injury with aspartate aminotransferase (AST) 102 U/L (grade 1) and ALT 223 U/L (grade 3), but no subjective symptoms, such as fatigue, itching, or anorexia.

The pharmacist confirmed that there were no additional medications other than the triplet regimen and kall, and that there was no alcohol or supplement use in his life history. The physician also responded that the triggers for the liver injury could be medications, not liver metastases. Therefore, the pharmacist reported to the physician the possibility caused by BINI and ENCO. In comparison with the previous blood draw, there were no changes in medications, except that the triplet regimen and kall were initiated. Therefore, the pharmacist reported to the physician that liver damage may have been caused by BINI and ENCO. In accordance with the dose adjustment criteria of the Japanese Guide for Appropriate Use, BINI and ENCO were discontinued; therefore, only CET was administered. Acetaminophen (AcA) was also temporarily withdrawn.

**Figure 3:**
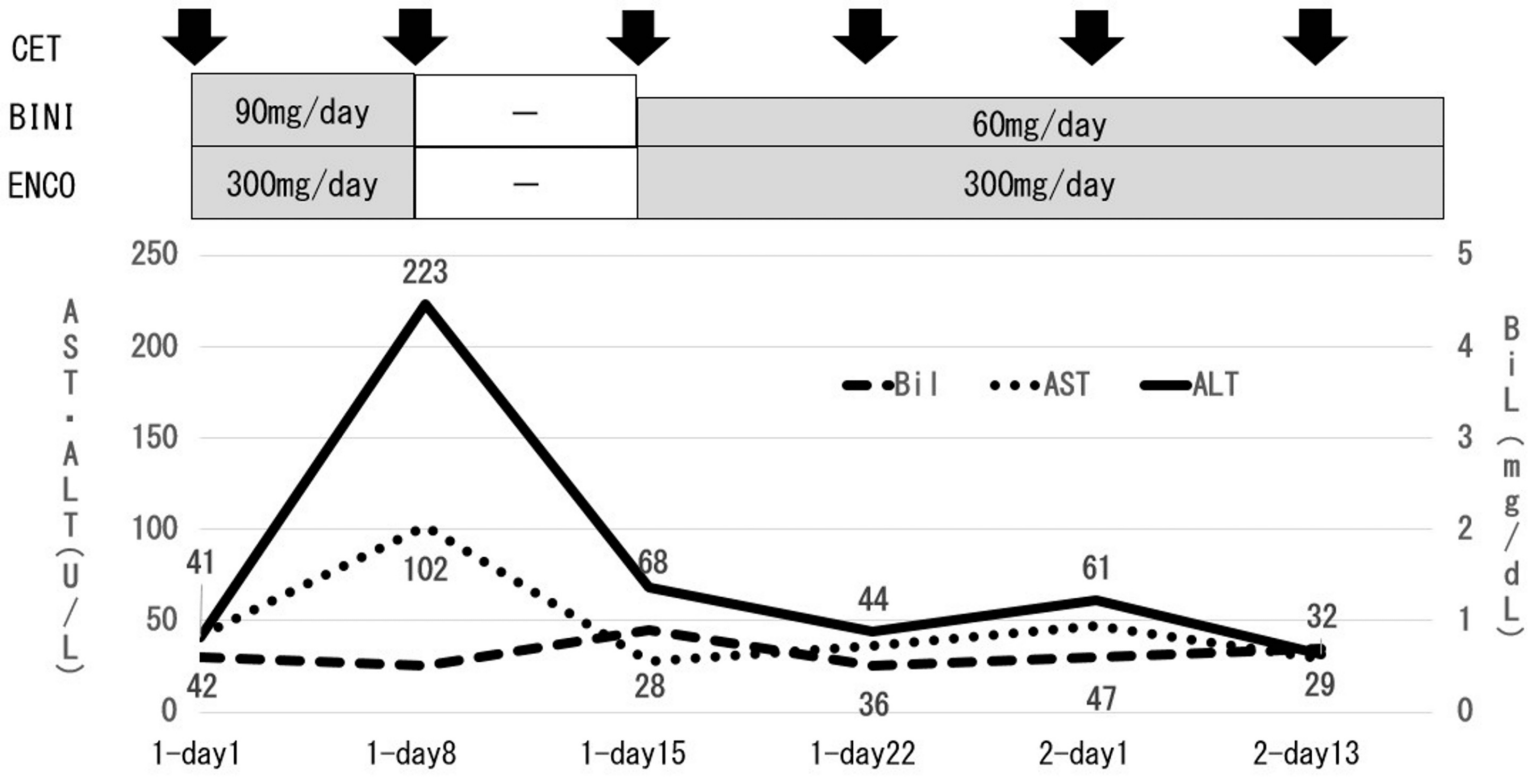
Clinical course of the patient. BINI: binimetinib; ENCO: encorafenib; AcA: acetaminophen; CET: cetuximab; AST: aspartate aminotransferase; ALT: alanine aminotransferase; Bil: bilirubin

On day 15, seven days after the discontinuation of BINI and ENCO, the attenuation of liver injury was noted with AST 28 U/L (grade 0) and ALT 68 U/L (grade 1). Therefore, BINI was restarted at a lower dose and ENCO at the same dose. The patient developed an acne-like skin rash (grade 2) and stomatitis (grade 2) and received medication for stomatitis. On day 22, liver injury showed further improvements with AST 36 U/L (grade 0) and ALT 44 U/L (grade 1); therefore, BINI and ENCO were continued at the same doses. The acne-like skin rash persisted at grade 2 and, thus, roxithromycin was prescribed. Treatment was continued after the second cycle without worsening of liver injury (Figure 3).

Drugs used were as follows: CET 460 mg (250 mg/m^2^), dexamethasone (dex) 6.6 mg (premedication), chlorpheniramine (chlo) 5 mg (premedication), BINI 50 mg 6C 1×, ENCO 15 mg 6T 2×, AcA 500 mg 4T 4×, esomeprazole (EPZ) 20 mg 1C 1×, loxoprofen (loxo) 60 mg 3T 3× (with self-regulation), and kall 25 units 6T 3×. The Naranjo adverse drug reaction probability score indicated that item 8 gave +1 for BINI and 0 for ENCO, with BINI receiving the maximum score (Table 2) [3].

**Table 2:** Naranjo score applied to the present case. The reaction is considered definite for a score ≥9, probable for 5-8, possible for 1-4, and doubtful for ≤0. A score of 10 out of 13 was obtained for BINI. Causality was considered definite. Therefore, BINI was withdrawn. BINI: binimetinib; ENCO: encorafenib; AcA: acetaminophen; CET: cetuximab; kall: kallidinogenase; chlo: chlorpheniramine; EPZ: esomeprazole; loxo: loxoprofen; dex: dexamethasone

Questions	Yes	No	Do not know	BINI	ENCO	AcA	CET	kall	chlo	EPZ	loxo	dex
1. Are there previous conclusive reports on these reactions?	1	0	0	1	1	1	1	1	0	1	1	0
2. Did the adverse event appear after the suspected drug was administered?	2	-1	0	2	2	2	2	2	2	2	2	2
3. Did the adverse reaction improve when the drug was discontinued or a specific antagonist was administered?	1	0	0	1	1	1	0	0	0	0	0	0
4. Did the adverse reaction reappear when the drug was readministered?	2	-1	0	2	2	0	-1	0	0	-1	-1	-1
5. Are there alternative causes (other than the drug) that could on their own have caused the reaction?	-1	2	0	2	2	2	2	2	2	2	2	2
6. Did the reaction reappear when a placebo was given?	-1	1	0	0	0	0	0	0	0	0	0	0
7. Was the drug detected in the blood (or other fluids) in concentrations known to be toxic?	1	0	0	0	0	0	0	0	0	0	0	0
8. Was the reaction more severe when the dose was increased, or less severe when the dose was decreased?	1	0	0	1	0	1	1	0	0	0	0	0
9. Did the patient have a similar reaction to the same or similar drugs in any previous exposure?	1	0	0	0	0	0	0	0	0	0	0	0
10. Was the adverse event confirmed by any objective evidence?	1	0	0	1	1	1	1	1	1	1	1	1
Total score	-	-	-	10	9	8	6	6	5	5	5	4
Interpretation of scores	-	-	-	Definite	Definite	Possible	Possible	Possible	Possible	Possible	Possible	Probable

## Discussion

We encountered an ALT elevation (grade 3) requiring drug withdrawal on day eight after the initiation of the triplet regimen. In an international phase III study, hepatotoxicity occurred within three months in 45.9% of cases (222/484 cases) [1], no specific timing of onset was reported in post-marketing surveillance [2], and the protocol did not specify a blood biochemical test on day eight [4]. Since laboratory findings are used to diagnose hepatic injury, severe hepatic injury may have occurred if the drug had been continued after day eight without laboratory testing [5].

The BRAF V600E mutation in colorectal cancer is detected in approximately 8-12% of patients and the prognosis is extremely poor [6]. It is generally more frequent in patients older than 60 years, in females, and in the right-sided colon, with a higher frequency of poorly differentiated carcinomas and mucous components [7]. However, our patient was a young male in his 40s with sigmoid colon adenocarcinoma and no family history. BRAF mutation-positive colorectal cancer is considered to be associated with the mitogen-activated protein kinase (MAPK) pathway for cancer growth. BINI and ENCO have been shown to promote epidermal growth factor receptor (EGFR)-mediated cell proliferation by deactivating the MAPK pathway and tumor growth is inhibited by the co-administration of CET [8].

All nine drugs administered in the present case were examined for their potential to induce adverse drug reactions. The Naranjo score was developed to standardize the assessment of causality for all adverse drug reactions and establish a causal association between a drug and an adverse event [3]. It consists of 10 questions that are answered as “yes,” “no,” or “do not know.” Different point values (-1, 0, +1, or +2) are assigned to each answer. Total scores range from -4 to +13; the reaction is considered definite for a score ≥9, probable for 5-8, possible for 1-4, and doubtful for ≤0. Table 1 shows that BINI and ENCO were highly relevant. However, item eight (“Were symptoms exacerbated when the dose was increased or alleviated when the dose was decreased?”) was rated as +1 for BINI and 0 for ENCO, resulting in a difference in scores. In clinical trials, the incidence of liver injury increased from 7.8% (17/216 patients) to 10.4% (23/222 patients) in the triplet regimen with BINI, which was initiated after the ENCO+CET combination [4]. Hepatic injury did not require drug withdrawal after the resumption of BINI with a dose reduction, implicating BINI. Therefore, hepatic injury was most likely related to BINI because it was not withdrawn after a dose reduction.

The main metabolic pathways for both drugs are hepatic metabolism - glucuronidation by UGT1A1 for BINI and CYP3A4 for ENCO [9]. Therefore, hepatic toxicity may be caused by the direct inhibitory effects of these drugs. Hepatic impairment, particularly when accompanied by elevated T-BIL, needs to be considered.

Adverse events other than hepatic injury in the present case were visual impairment and stomatitis (both grade 2) as well as an acne-like skin rash (grade 2). However, the patient was able to continue treatment without the worsening of symptoms with supportive care.

Progression-free survival (PFS) with the triplet regimen was previously reported to be 4.3 months. In the present case, the triplet regimen was withdrawn for seven days starting from day eight. PFS was approximately 5.5 months, which was consistent with previous findings. Although the triplet regimen is expected to have a limited therapeutic effect, most adverse events occur early, within one to three months, and are reversible [1]. Therefore, the monitoring of adverse events immediately after induction is considered to be important for the continuation of treatment.

## Conclusions

We encountered an ALT elevation (grade 3) requiring drug withdrawal on day eight after the initiation of the triplet regimen. Therefore, biochemical testing on day eight of the triplet regimen is important for the safe continuation of drug therapy. Further investigations are needed to establish the specific timing of hepatic injury and accumulated cases because this approach is expected to be used more frequently as a second-line treatment for BRAF mutation-positive colorectal cancer.
